# Supplementary material for: Dissociable consequences of moderate and high volume stress are mediated by the differential energetic demands of stress
Source: PLoS One. 2022 Sep 1;17(9):e0273803. doi: 10.1371/journal.pone.0273803 (PMC9436037; doi:10.1371/journal.pone.0273803)
Supplement: Attachment — Submitted filename: reviewer_comments_v2.docx [file pone.0273803.s001.docx]

When submitting your revision, we need you to address these additional requirements.

1. Please ensure that your manuscript meets PLOS ONE's style requirements, including those for file naming. The PLOS ONE style templates can be found at

https://journals.plos.org/plosone/s/file?id=wjVg/PLOSOne_formatting_sample_main_body.pdf and

https://journals.plos.org/plosone/s/file?id=ba62/PLOSOne_formatting_sample_title_authors_affiliations.pdf

2. Thank you for stating the following in the Acknowledgments Section of your manuscript:

"This research was supported by NIH Grant R01-MH115678 and funds from the Staglin Center for Brain & Behavioral Health."

We note that you have provided funding information that is not currently declared in your Funding Statement. However, funding information should not appear in the Acknowledgments section or other areas of your manuscript. We will only publish funding information present in the Funding Statement section of the online submission form.

Please remove any funding-related text from the manuscript and let us know how you would like to update your Funding Statement. Currently, your Funding Statement reads as follows:

"This research was supported by NIH Grant R01-MH115678 (M.S.F.) and funds from the Staglin Center for Brain & Behavioral Health (M.S.F.). The funders had no role in study design, data collection and analysis, decision to publish, or preparation of the manuscript."

Please include your amended statements within your cover letter; we will change the online submission form on your behalf.

The above funding statement is correct. As such there are no changes necessary. For financial disclosures, however, we would like to add “Financial Disclosures-MSF is a board member of Neurovation Inc. The authors declare that the research was conducted in the absence of any commercial or financial relationships that could be construed as a potential conflict of interest.” The acknowledgment and financial disclosure sections have been removed, per request, to avoid redundancy.

3. In your Data Availability statement, you have not specified where the minimal data set underlying the results described in your manuscript can be found. PLOS defines a study's minimal data set as the underlying data used to reach the conclusions drawn in the manuscript and any additional data required to replicate the reported study findings in their entirety. All PLOS journals require that the minimal data set be made fully available. For more information about our data policy, please see http://journals.plos.org/plosone/s/data-availability.

Upon re-submitting your revised manuscript, please upload your study’s minimal underlying data set as either Supporting Information files or to a stable, public repository and include the relevant URLs, DOIs, or accession numbers within your revised cover letter. For a list of acceptable repositories, please see http://journals.plos.org/plosone/s/data-availability#loc-recommended-repositories. Any potentially identifying patient information must be fully anonymized.

It appears we failed to note the github link somewhere within the submission, our apologies. It is available at <https://github.com/mconoscenti/shock_volume>. This has also been noted in the revised cover letter.

Important: If there are ethical or legal restrictions to sharing your data publicly, please explain these restrictions in detail. Please see our guidelines for more information on what we consider unacceptable restrictions to publicly sharing data: http://journals.plos.org/plosone/s/data-availability#loc-unacceptable-data-access-restrictions. Note that it is not acceptable for the authors to be the sole named individuals responsible for ensuring data access.

We will update your Data Availability statement to reflect the information you provide in your cover letter.

4. PLOS ONE now requires that authors provide the original uncropped and unadjusted images underlying all blot or gel results reported in a submission’s figures or Supporting Information files. This policy and the journal’s other requirements for blot/gel reporting and figure preparation are described in detail at https://journals.plos.org/plosone/s/figures#loc-blot-and-gel-reporting-requirements and https://journals.plos.org/plosone/s/figures#loc-preparing-figures-from-image-files. When you submit your revised manuscript, please ensure that your figures adhere fully to these guidelines and provide the original underlying images for all blot or gel data reported in your submission. See the following link for instructions on providing the original image data: https://journals.plos.org/plosone/s/figures#loc-original-images-for-blots-and-gels.

In your cover letter, please note whether your blot/gel image data are in Supporting Information or posted at a public data repository, provide the repository URL if relevant, and provide specific details as to which raw blot/gel images, if any, are not available. Email us at plosone@plos.org if you have any questions.

The raw Western Blot data has been uploaded to Github as well, <https://github.com/mconoscenti/shock_volume/tree/mconoscenti-westernblot-raw>.

[Note: HTML markup is below. Please do not edit.]

Reviewers' comments:

Reviewer's Responses to Questions

Comments to the Author

Reviewer #1: The manuscript reports a series of experiments examining how “shock volume” impacts glucose-mediated fear learning. In the manuscript, the authors contrast three groups: High-volume shock (HVS; 100 shocks), moderate-volume shock (MVS; 15 shocks) and no-shock controls (NS). In the first experiment there was a double dissociation between shock volume and contextual fear and weight gain. MVS increased contextual fear and had no impact on weight gain, whereas HVS had no impact on fear but decreased weight gain. Both HVS and MVS increased expression of auditory fear conditioning. Next, rats that consumed a glucose solution also showed enhanced contextual fear relative to NS, and glucosprivated rats did not show enhanced contextual fear following MVS. Thus, the authors show bi-directional control of this the impact of MVS on contextual fear. Finally, the authors report dissociable effects of glutamate receptor expression in the BLA and DH.

Overall, this is an excellent series of experiments. As mentioned earlier, it is particularly thoughtful to see effects manipulated in both directions. I have one major comment, and several more minor comments.

Major: My main concern is related to the cued fear conditioning experiment. The was a nice addition; it allowed the authors to examine if the MVS vs. HVS dissociation was general to fear expression or specific to the expression of contextual fear learning. In this experiment, while MVS increased contextual fear, and HVS did not (relative to NS), expression of cued fear was increased for both MVS and HVS.

The concern I have is that the authors describe the tone-shock association as not requiring the hippocampus. While it is certainly true that there are many reports that delay fear conditioning is not impacted by pre- or post-training damage to the hippocampus, there is additional research that suggests this may depend on the “strength” of the association. Indeed, Quinn et al. (2008) demonstrate that when the tone-shock association is weak, then expression of auditory fear conditioning does depend on the hippocampus. Given that in the current paper there was only one pairing of the tone and shock, it seems reasonable that this would be considered a weak association.

I’m sure the authors are aware of this, and likely have some thoughts. I don’t think this needs to be addressed with additional experiments. But I wonder if the authors could address this issue. Although it would be a strong argument if indeed this tone-shock association were hippocampal independent, I do think it is still a compelling dissociation.

This really is an interesting worth acknowledgement in the paper. It is true that out single-trial conditioning would reasonably fall within a procedure that produces a weak association. Indeed, that’s why the procedure is chosen! A weak association allows for very clear SEFL effects. However, in a sufficiently stressed animal, a single trial no longer produces a weak association. In the 100 shock group, however, since they are not exhibiting high levels of contextual freezing, it becomes a much larger puzzle to determine if our single-trial conditioning should be considered a procedure that nourishes a weak or strong association.

In an effort to remain succinct within the manuscript, we have added a note regarding the Quinn et al (2008) series of experiments, but have not gone into the weeds regarding this issue. Note that in the Quinn study hippocampal lesion effects were typically seen after, not during CS presentation, the data we presented were all during CS presentation. We have also softened claims suggesting that this experiment comprehensively tests this hypothesis.

Minor:

1) Pg. 7. The experiments were run in only male rats. However, the authors provide suitable logic for this in the general discussion.

Thank you for noting this important point.

2) All the figures were “fuzzy”. This might have been an issue with my own printer, or maybe with the pre-processing by the journal. I only mention it just so the authors are aware in case it is related to the resolution they were saved as.

Thank you for pointing this out. The files were uploaded at high resolution, but we will be sure that the figures look clear during the preprint process.

3) Pg. 8 and 9. In general the reader could use some more details regarding the procedures of the experiment. As just one example, the authors note that rats were pre-exposed to a novel context, but they don’t mention duration of exposure.

Information regarding preexposure has been added. Other clarifying language regarding procedures and reorganization of the method section has been performed to better spell out all procedures performed.

4) Pg. 11. The authors report an ANOVA on post-shock freezing. But I think they are referring to the retrieval test?

Yes, that is correct. Wording has been changed to accurately reflect this.

5) Pg. 11. The results for figure 1 might be re-ordered. For the reader, it might be more logical to discuss panel A then B then C then D. I thought this was also true for the way the data is described for Figure 2.

This point is appreciated. We have reordered the results of B & D to read in the appropriate order. We have kept the discussion of A & C together, however, to avoid seeming unnecessarily redundant by discussing the results of 1-day and 1-week separately where they are nearly identical.

6) I like the additional analysis of shock reactivity and baseline freezing. I think the authors can decide – but would it be worth it to include in the figures?

These data were included in initial internal drafts. However, because much of the baseline freezing data was zero, it felt unnecessary to provide graphs that were essentially empty.

7) For brevity, when an F value is less than 1, I think the authors could report (F < 1) as opposed to the full value (e.g, F = 0.006837).

Where applicable, the full reporting has been changes to simply “F < 1”.

8) Figure 2 panel C. Trials on the X-axis. This this 1-minute time bins?

The x-axis denotes the three preexposure trials. Hopefully, with the additional information regarding our preexposure procedure, this becomes clearer to the reader. We have also included further description in the figure legend.

Reviewer #2: The present manuscript (PONE-D-22-09431) examines the impact of different doses of tailshock (15 vs 100 shock procedures) on subsequent fear conditioning and glutamate receptor protein expression. The authors state that the 15-shock procedure is analogous to the stress-enhanced fear learning model and the 100-shock procedure is analogous to the learned helplessness paradigm. The authors present a reasonable hypothesis that the two procedures generate different molecular and behavioral sequelae due to differences in metabolic demand.

The hypothesis would be strengthened if the authors provide a comparison of post-stress metabolic measures between the two stress types (moderate vs high-volume stress). Giving 2-deoxy-D-glucose is not the same as directly measuring energy homeostasis. Furthermore, do the authors have any evidence that the glucose manipulations that alter the behavioral phenotype also alter their hippocampal receptor subunit findings?

Post-stress metabolic measures would indeed help illuminate the potential mechanism of these effects. Indeed, we have previously reported that HVS greatly challenges metabolic homeostasis (Conoscenti, et al. 2019). However, these types of studies are extensive and we felt were beyond the purview of the current study. However, getting into the physiological mechanisms of the reported effects is an excellent future direction that has been added to the discussion section. We did indeed attempt to examine the effects of glucose on the receptor subunit findings in the same animals that underwent behavioral testing. We have found, however, that subsequent testing (ie. fear conditioning) shrouds the stress effects, and the data were therefore inconclusive.

One issue with framing the present results in the context of stress-enhanced fear learning (SEFL) versus learned helplessness (LH) is that the authors don’t accurately define what learned helplessness actually is. LH refers to stressor outcomes that depend on the uncontrollability of the stressor. That is, to qualify as a LH effect an outcome must follow exposure to inescapable (IS), but not physically identical escapable (ES), shocks or other aversive events. To ignore the controllability issue is to make LH effects synonymous with generic stress effects, which they are not. There are plenty of outcomes (e.g., neurochemical, behavioral) of tail shock that are not sensitive to the dimension of controllability. Thus, there can be many reasons for poor shuttlebox escape responding, only one of which is learned helplessness.

We initially reference *learned helplessness* not as a means to tie the current results with the phenomena, but merely to provide a justification for the stress procedure used. We agree that it is impossible to determine LH without the escapable group. However, we also know that the effect is somewhat unique in that it requires a significant exposure to (uncontrollable and unpredictable) stress. Whether that be 300-800 seconds of total shock, this is still far above the level of shock required for many other behavioral effects (such as SEFL). It therefore provides us with an excellent procedural comparison to more moderate stressors that do not produce these unique shuttle-escape deficit effects. To be clear, here we are looking at the effects that two types of stress have on behavior and are not exploring how control can reduce the effects of stress. Wording stressing this effect has been added to the introduction.

Additionally, we had previously attempted to separate LH from HVS as quickly as possible, while still acknowledging it as a source of inspiration. To extend this end, we have further refined this section of the introduction to further clarify that we are making no statements about LH *per se*, but rather interested in the 100 shock procedure, originally tied to LH, that produces an array of unique effects.

Related to above, the authors imply that the gold standard for LH experiments is to deliver 100 x 1.0 mA shocks for an average length of 8 seconds (800 seconds of total shock). The majority of published LH studies (those that include inescapable and escapable groups) use considerably less shock volume. Mean wheel-turn escape times across 100 shocks are around 3-5 seconds (300-500 seconds of total shock). If the authors frame their high-volume stress treatment as a typical learned helplessness design, then they should provide references demonstrating that their shock parameters produce effects that are selective to the uncontrollability of the stressor.

This point is noted and appreciated. The description of canonical shock duration has been adjusted to a range to reflect this point.

The claim that these results represent “the first study to demonstrate tailstock-to-footshock” stress-enhanced fear learning ignores a number of published papers showing that tail shock enhances fear learning (both cued and contextual). As an example, uncontrollable tail shock leads to enhanced footshock-elicited freezing in a shuttle box 24 hr later (plenty of studies from the Maier laboratory), a conditional response attributable to contextual cues of the shuttle box apparatus (Fanselow, 1980). In fact, the majority of studies show that uncontrollable tail shock increases, rather than interferes with, contextual fear.

The reviewer is absolutely right on this point. We were really intending this statement to be specific to the canonical SEFL design (15 shock stressor followed by 1-trial conditioning) in order to address previous critiques of the model. This has been reworded to clarify this claim.

The abstract states that weight gain was impacted only in high-volume stress animals. Was this a consistent finding throughout the studies? It appears that weight gain was depressed in both high and moderate groups compared to No Stress in Figure 3. And in Figure 4B the % weight change for the MVS-Veh group is similar to that of the HVS group in Figure 1D.

This finding was consistent across all studies (ie. see figure 5), except for the study where the glucose manipulation was present (though the general trend here is consistent). We did find it interesting in the case of the glucose study, and our best guess was that the preexposure to glucose provides its own (albeit opposite) challenge to energy homeostasis which may be somewhat hormetic to future challenges. This may be enough to obfuscate any stress effects on weight. This experiment also required several cohorts due to its size. Therefore it’s also possible that the variability between cohorts was enough to mitigate the statistical power between stress groups. As for Figure 4B, the control group suggests that this cohort may have generally been slower at gaining weight.

[NOTE: If reviewer comments were submitted as an attachment file, they will be attached to this email and accessible via the submission site. Please log into your account, locate the manuscript record, and check for the action link "View Attachments". If this link does not appear, there are no attachment files.]

While revising your submission, please upload your figure files to the Preflight Analysis and Conversion Engine (PACE) digital diagnostic tool, https://pacev2.apexcovantage.com/. PACE helps ensure that figures meet PLOS requirements. To use PACE, you must first register as a user. Registration is free. Then, login and navigate to the UPLOAD tab, where you will find detailed instructions on how to use the tool. If you encounter any issues or have any questions when using PACE, please email PLOS at figures@plos.org. Please note that Supporting Information files do not need this step.
