# Supplementary material for: Dissociable consequences of moderate and high volume stress are mediated by the differential energetic demands of stress
Source: PLoS One. 2022 Sep 1;17(9):e0273803. doi: 10.1371/journal.pone.0273803 (PMC9436037; doi:10.1371/journal.pone.0273803)
Supplement: Attachment — Submitted filename: reviewer_comments_secondround.docx [file pone.0273803.s002.docx]

The authors still use the term “learned helplessness” in a way that is synonymous with “stress”. The term is not appropriate for simply any behavioral or physiological consequence of uncontrollable tailshock. Rather, it is appropriate for behavioral changes that are specifically mediated by the inescapable/uncontrollable nature of the shock, and not the shock itself. The distinction is important since the motivation of the present experiments (Intro), the hypothesis (line 171), and experimental design (line 208) are framed within the context of LH.
Statements such as “Rats exposed to LH experience a total of about 800 seconds of 1 mA shock (100 shocks at an average of 8 seconds each)” and “HVS and MVS parameters were chosen to mimic previously published work on LH…” are not supported by any reference that shows that these parameters produce learned helplessness. Certainly they produce stress effects, but not necessarily LH.

- We have further clarified this point. As stated before, it is not our intention to draw conclusions regarding learned helplessness, or draw a misleading premise. Specifically, we have added language in the into to reinforce the point that the HVS stress is simply a stressor that is parametrically similar to the LH-inducing stressor. We have also added clarifying language to the hypothesis line and experimental design to avoid any further confusion on this point.
